# Supreme Nasal Turbinate as an Additional Surgical Landmark in Endoscopic Sinus and Skull Base Surgeries

**DOI:** 10.7759/cureus.8132

**Published:** 2020-05-15

**Authors:** Siti Nazira Abdullah, Baharudin Abdullah

**Affiliations:** 1 Otolaryngology, School of Medical Sciences, Universiti Sains Malaysia, Kubang Kerian, MYS

**Keywords:** supreme nasal turbinate, endoscopic sinus surgery, endoscopic skull base surgery

## Abstract

During endoscopic sinus and skull base surgeries, surgical landmarks are routinely used to guide surgeons navigating in the narrow corridor of the sinonasal region. Risk of complications is higher in difficult cases when there is excessive bleeding or alteration of the normal anatomical landmarks by tumour. An additional landmark is advantageous to prevent complications and serves as a guide. We present a case of supreme turbinate found incidentally during an endoscopic transsphenoidal surgery. Not much is known about the role of supreme turbinate. When it is present, the sphenoid ostium is located medial to its posteroinferior attachment, and behind its vertical part. Hence, the identification of this structure serves as an additional landmark besides superior turbinate during surgery.

## Introduction

In endoscopic sinus and skull base surgeries (ESSBS), anatomical landmarks are useful to ensure proper identification of sinonasal structures, allow precise dissection and avoid complications. Structures such as middle turbinate (MT), orbital floor and superior turbinate (ST) are recognized as important surgical landmarks in ESSBS [1]. Sphenoid sinus may need to be cleared of disease or as an access to remove tumour at the skull base. But identification of the sphenoid ostium (SO) during ESSBS may be difficult to beginners or may be challenging especially when the normal structures are destroyed or involved in any pathological condition. One of the methods to identify the SO is by first locating the ST as the anatomical landmark and from there the ostium may be found medial to it. In an inflammatory polyposis or sinonasal tumour, when there are excessive bleeding and distortion of the normal anatomy, an additional landmark is a good safeguard for safer surgery. Supreme turbinate (SupT) may serve as an additional landmark besides the ST in ESSBS, especially when the ST is destroyed by disease or when it has to be removed due to tumour involvement. In this case presentation, we present our experience in utilizing the SupT as a surgical landmark while performing an endoscopic skull base surgery.

## Case presentation

**Figure 1:**
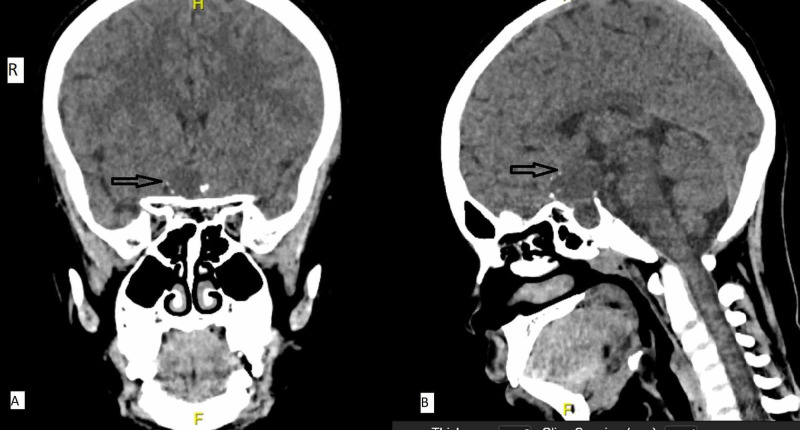
Computed tomography of the brain (A, coronal image and B, sagittal image) shows the tumour located superior to the pituitary gland (arrow).

A 14-year-old boy with a craniopharyngioma (Figure 1) underwent an endoscopic transsphenoidal surgery for tumour removal.

**Figure 2:**
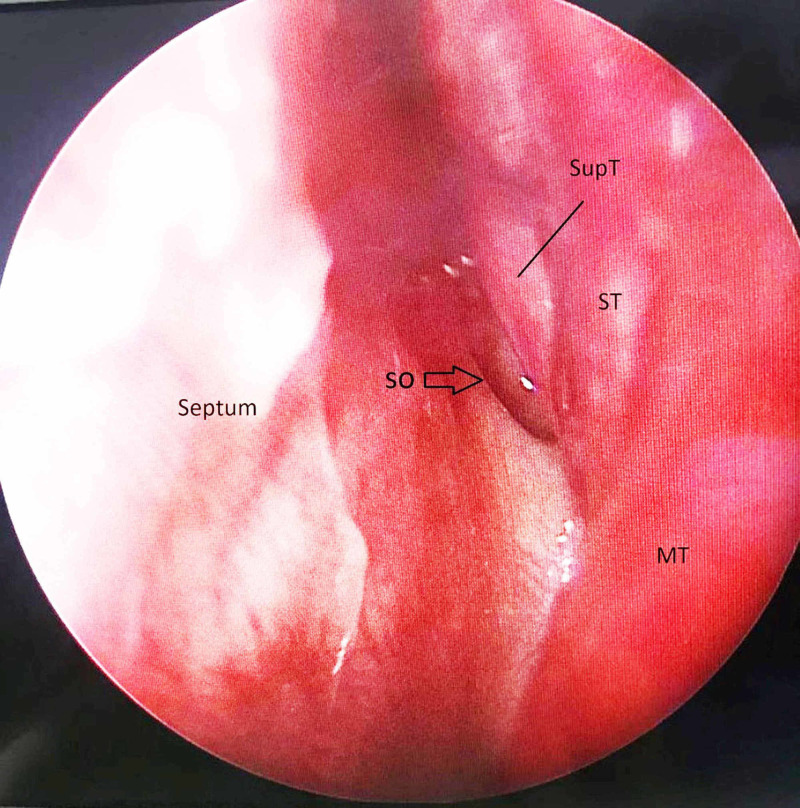
Nasoendoscopic view of the relation of the left supreme turbinate to both the left sphenoid ostium and left superior turbinate. SupT-supreme turbinate, MT-middle turbinate, ST-superior turbinate, SO-sphenoid ostium

Intraoperatively, a 4 mm 0-degree Hopkins rigid scope was inserted into the left nasal cavity and the presence of a SupT was observed, medial to the left MT and left ST but lateral to the left SO (Figure 2).

The left SO was opened and enlarged in an inferolateral direction. The lower one-third of the left ST was resected. The left SupT was left intact. Similarly, the right SO was opened and joined with the left sphenoid to create a wide opening of the common sphenoid sinus cavity. Further dissection was done as per the usual steps for transsphenoidal surgery. The tumour was removed successfully, and no complications were encountered postoperatively.

## Discussion

Due to the complex anatomy of the sinonasal structure, anatomical variations, narrow surgical field and poor handling of instruments in unskilled hands, inadvertent complications may occur in ESSBS. The use of navigation system has significantly reduced the complications and improved the outcome. But it is not a substitute for sound surgical judgement and anatomical knowledge [2]. When surgeons do not have sufficient understanding of the anatomy to navigate their way safely during ESSBS, dissection becomes difficult and potentially hazardous.

Not much is known about the role of SupT. When it is present, the SO is located medial to its posteroinferior attachment, and behind its vertical part. Consequently, the identification of this structure serves as an additional landmark besides ST during ESSBS. SupT has a prevalence of 60% and may present unilaterally or bilaterally [3]. But depending on each specific population, a prevalence rate as high as 77% has been reported [4]. It is a variant of the primary turbinates, consisting of superior, middle and inferior bony projections from the lateral nasal wall [5]. It should not be considered as a secondary ST; the ethmoid bulla and uncinate process are considered as secondary turbinates while secondary MT is a variant secondary turbinate [6]. Neither should it be seen as an accessory ST, such as the case of an accessory MT, which is actually a medially bent uncinate process, as it has its own attachment [7]. Moreover, it has to be properly recognized as an anatomical variant and not to be confused as an abnormal mass in order to avoid unnecessary investigation and surgery.

## Conclusions

SupT is an anatomical variant that is useful as an additional surgical landmark in ESSBS. The additional surgical landmark may be required when the usual anatomical structure is already resected or destroyed by disease. It is also useful when there is excessive bleeding where surgeons may need additional confirmation.
